# Transforming Seven Kinds of Relational Pain

**DOI:** 10.1111/jmft.70043

**Published:** 2025-06-16

**Authors:** Joaquín Gaete‐Silva, Karl Tomm, Inés Sametband, Sumerlee Samuels

**Affiliations:** ^1^ University of Calgary Calgary Alberta Canada; ^2^ Calgary Family Therapy Centre Calgary Alberta Canada; ^3^ Department of Psychology Mount Royal University Calgary Alberta Canada

**Keywords:** family therapy, interaction patterns, interpersonal conflict, IPscope, reflexive questions, relational pain, relational preferences

## Abstract

Bringforthist therapy extends the systemic practices of Interventive Interviewing and applies the relational focus of the IPscope. We elaborate upon these frameworks, highlighting their sensitivity to locally shaped needs of family members through the notions of *relational preferences* and *relational pain*. Relational preferences refer to family members' normative expectations about their relationships (relationship “shoulds”), while relational pain refers to family members' experiences of unfulfilled hopes vis‐à‐vis their culturally situated relational preferences (relationship “can'ts”). We distinguish seven variations of unrealized preferences, which are aggravated by differing pathologizing patterns of interpersonal interaction. We label these as seven “M's” of relational pain: Misjudging, Misaligning, Misrecognizing, Misappropriating, Mistreating, Mistrusting, and Misgrieving. We discuss each of these in relation to seven corresponding “R's” that could bring forth relational healing, namely Reappraising, Realigning, Recognizing, Reappropriating, Revealing, Reconciling, and Re‐membering. In addition, we outline prototypical interaction patterns to illustrate how these different forms of pain might be addressed.

Therapists informed by Bringforthist Therapy (Tomm et al. 2025) envision individuals responding to significant others in patterned interactions that *bring forth* their social worlds (e.g., values, roles, practices) which, recursively, brings them forth as embedded participants in ongoing relationships. This interactive understanding extends to the relationship between a therapist and a family as well as to the relationships among family members. As such, instead of imposing normative views on how to be a good family, Bringforthist therapists prioritize therapeutic practices that are attuned to each family's unique forms of relational pain, which we see as influenced by locally shaped relationship hopes.

## 1 Relational Pain = Hope + Can't

People usually come to couple and family therapy to address some kind of relationship difficulty; something has happened or is happening in their relationship(s) that is hurting them deeply. We specify such pain as relational to distinguish it from physical pain and delineate it as the type of discomfort that motivates people to seek relational forms of therapy (e.g., family therapy, psychotherapy) rather than physiotherapy. Sometimes the kinds of pain that arise from relational injuries are referred to as mental pain or emotional pain. This construal tends to privilege an individualizing focus in understanding human experience. Although the pain is felt by individuals, we refer to it as relational pain to emphasize that (a) it arises from relationships with someone or something they care deeply about (e.g., culturally shaped values); and (b) individuals understand their pain as involving relationship difficulties as obstacles to realize their hopes.

Take the situation of a parent who seeks therapy with a concern about their child being too aggressive. For the aggression to be experienced as relationally painful, it requires (a) being evaluated against an implicit hope such as “people should behave respectfully, in prosocial ways.” In other words, when someone notices a discrepancy between a relational hope (e.g., a normative expectation that a child should be respectful toward others) and the actual experience (e.g., “my child is very rude, and too aggressive”), they will likely experience relational pain. For this pain to become a candidate for relational therapy, we imagine that the parent has some pre‐figurative understanding about (b) some yet‐to‐be‐known relational barriers that may be getting in the way of the child behaving prosocially (e.g., “my child doesn't listen to me, maybe they'll open up to you and listen… perhaps they could develop some skill…. or I could learn strategies to help them treat others respectfully”).

The relational aspects of mental pain are often minimized in the culturally dominant ways of understanding pain and suffering within the mental health professions. For example, in psychiatric manuals such as the DSM, mental pain is often regarded as a symptom of an underlying syndrome or mental disorder (American Psychiatric Association 2013). In some circles, this discourse promotes conceptions of mental health concerns such as conduct problems (e.g., aggression, defiance, cheating, lying, self‐harm, and suicidality) or emotional problems (e.g., anxiety, depression, gender identity issues, and trauma‐related issues) as something existing under a person's skin that needs to be fixed—“a clinically significant disturbance… that reflects a dysfunction in the psychological, biological, or developmental processes underlying mental functioning” in an individual (American Psychiatric Association 2013, p. 14). Such formulations tend to overlook that these (so‐called) symptoms‐of‐individual‐dysfunction often become disruptive or painful to others (Gaete‐Silva and Gaete 2021). Moreover, those who are disrupted often respond in judgmental ways that beget more pain in others—and more of the disruptive behavior. By bringing forth such pain as relational, we hope to provide a systemic lens to appreciate both how the concept of pain may be formulated as interpersonal and how such pain can be maintained, or dismantled, through particular forms of relating.

We see relational pain as arising from an experience of not being adequately loved, being ignored, demeaned, or negated by significant others. Paraphrasing Maturana, life hurts in places where you are not seen (Amiguet 2005). At an experiential level, several types of relational pain can be linguistically expressed using sentences in the form of “I can't …” (Gaete and Gaete 2021), implying that there is a hope involving significant others, which cannot be realized. Since the “I can't” involves unfulfilled desires within significant relationships, we infer an underlying “relational hope,” a strong preference for something better in the relationship rather than some trivial want. These hopes can be seen as culturally situated, normative, ethical assessments vis‐à‐vis how people aspire to live their lives (Gaete, Sametband, et al. 2018; Taylor 1985); something people feel they “should” have, do, or be, in relation to significant others. Of course, since individuals frequently internalize or adopt significant others' perspectives regarding themselves, relational hopes may underlie people's pain without a need of the actual presence of the other. The pain arises when we perceive a discrepancy between our relational “shoulds” and our powers to realize them. Simply put then, *relational pain = hope + can't*.

Naming a problematic behavior, such as aggression, does not tell us much about what might be prompting the aggressive behavior. It could be that a child is displaying aggression (or “*can't* be as prosocial as they should”) because they feel oppressed by something or someone. Or it could be that the child is experiencing an inconsolable loss. Alternatively, the aggression may be the result of a lack of opportunities to learn how to manage frustration and regulate their emotions. Or perhaps the aggression is the result of some form of abuse, which has not been addressed.

Instead of labeling different kinds of problematic behaviors, syndromes, or mental disorders, in this article, we distinguish seven forms of underlying relational pain. We do not see these seven as exhaustive; we simply offer them as common presentations of pain in our context of therapeutic practice. Our understanding of pain as relational is a mainstay of the Bringforthist therapy as developed at the Calgary Family Therapy Centre. We have come to see the naming of these pains as marking opportunities to bring forth differing patterns of therapeutic practice to address each of them specifically. Two important frameworks that support this process in Bringforthist therapy are Interventive Interviewing and the IPscope. We briefly review each framework to provide a context for some of our recent practices in deconstructing relational pain and co‐constructing relational wellness.

## 2 Interventive Interviewing and Reflexive Questioning

In a series of papers on Interventive Interviewing, Tomm (e.g., 1987) suggested that certain questions could be therapeutic not because they generate insight among family members but because they help draw reflexive distinctions regarding relational hopes. These distinctions provide ways of understanding their relationships that not only depict but also shape them in preferred ways, enabling family members “to generate or generalize constructive patterns of cognition and behavior on their own” (1987, p. 4).

To illustrate reflexive questioning, Tomm (1987) reported on a therapy session where a family presented with several children complaining incessantly about their father being too aggressive in his parenting. The therapist posed a reflexive question to the children about the effects that a hypothetical absence of their mother might have on the relationship with their so‐called tyrannical father. The question activated the children's imagination (e.g., “he'd probably help us with cooking and homework”), then ad‐hoc memories from their past relationship (e.g., “like the time when he…”), and finally current attitudes of seeing their father as affectionate, to the point that the children began defending him. According to Tomm (1987), the reflexive question ended up “interrupting the malignant process of blame [enabling] the children to ‘bring forth’ a construal of their father as a caring parent” (p. 1). This appeared to have a healing effect as it opened space to realize a preferred way for the children to relate to their father.

Tomm (1987) referred to these *reflexive questions* as interventive, that is, as being “asked with the intent to facilitate self‐healing” (p. 4) within the family system. To avoid speculating on therapists' intentions, Gaete, Couture, et al. (2018) more recently redefined reflexive questions in terms of their observable relational effects. Specifically, these questions enable the co‐construction of preferred understandings of relationships among family members (e.g., nurturing parenting as preferred over tyrannizing parenting). In other words, reflexive questions could help clients change the viewing and doing of their relationships in mutually preferred ways, thereby transforming relational pain into relational wellness (Gaete, Sametband, et al. 2018, 2021).

## 3 The IPscope (PIPs, HIPs, WIPs, TIPs, SCIPs and DIPs)

Supplementing the interventive interviewing framework, Tomm et al. (2014) developed the IPscope (Interpersonal‐Pattern‐scope) to assess self‐sustaining relational influences or *Interpersonal Patterns* (IPs), which are constituted by mutual invitations that maintain either relational pain or relational wellness. What turns these invitations into self‐sustaining patterns is that they form mutually triggering couplings. For instance, a child's blaming practices may trigger a father's denying practices, which in turn trigger more blaming, consequently triggering more denying and so forth, thereby establishing and maintaining a *Pathologizing Interpersonal Pattern* (PIP) rooted in the malignant process of blaming. Such interaction patterns of *blaming coupled with denying* are seen as perpetuating the relational pain (Tomm et al. 2014).

In contrast to such negative patterns, *acknowledging practices coupled with appreciative practices* constitute a positive *Healing Interpersonal Pattern* (HIP), which could provide a kind of antidote to the relational pain of the abovementioned PIP. These respective Interpersonal Patterns (IPs) could be further stabilized by becoming coupled with relational hopes shaped by locally powerful ideas or *Socio‐Cultural Interpersonal Patterns* (SCIPs) that feed into such practices. For instance, an expectation such as “fathers should not be tyrannical with their children” could activate objections to fathering practices distinguished/experienced as tyrannical, thus intensifying the judgments feeding a family's *blaming coupled with denying* PIP and amplifying the relational pain that comes with it. On the other hand, positioning the father as “caring” in a manner that fits local relational hopes may feed into a family's *acknowledging coupled with appreciating* HIP. In time, this HIP may become further stabilized and evolve into a broader manner of interacting that more fully realizes the family's relational hopes, that is, a *Wellness Interpersonal Pattern* or WIP, such as *warm parental nurturing coupled with children honoring the parental supportiveness*.

Reflexive questioning itself can be used to interrupt painful relational patterns (e.g., PIPs), thus opening space for the emergence of preferred patterns (i.e., HIPs and WIPs). In applying the IPscope framework, the interactive process of interrupting painful PIPs and/or promoting relief through HIPs is distinguished as a TIP or *Transforming Interpersonal Pattern* (Tomm et al. 2014). A generic TIP in Bringforthist therapy may be described as a *therapist asking reflexive questions coupled with family members clarifying* preferred ways of relating (which could facilitate family members re‐coordinating in such preferred ways—thus engaging in a HIP). From this perspective, TIPs become a hallmark of conversational processes that can be called therapeutic. Accordingly, TIPs have been defined as relationship patterns whereby therapists and clients coordinate the family's relational hopes (Gaete et al. 2021).

**Figure 1:**
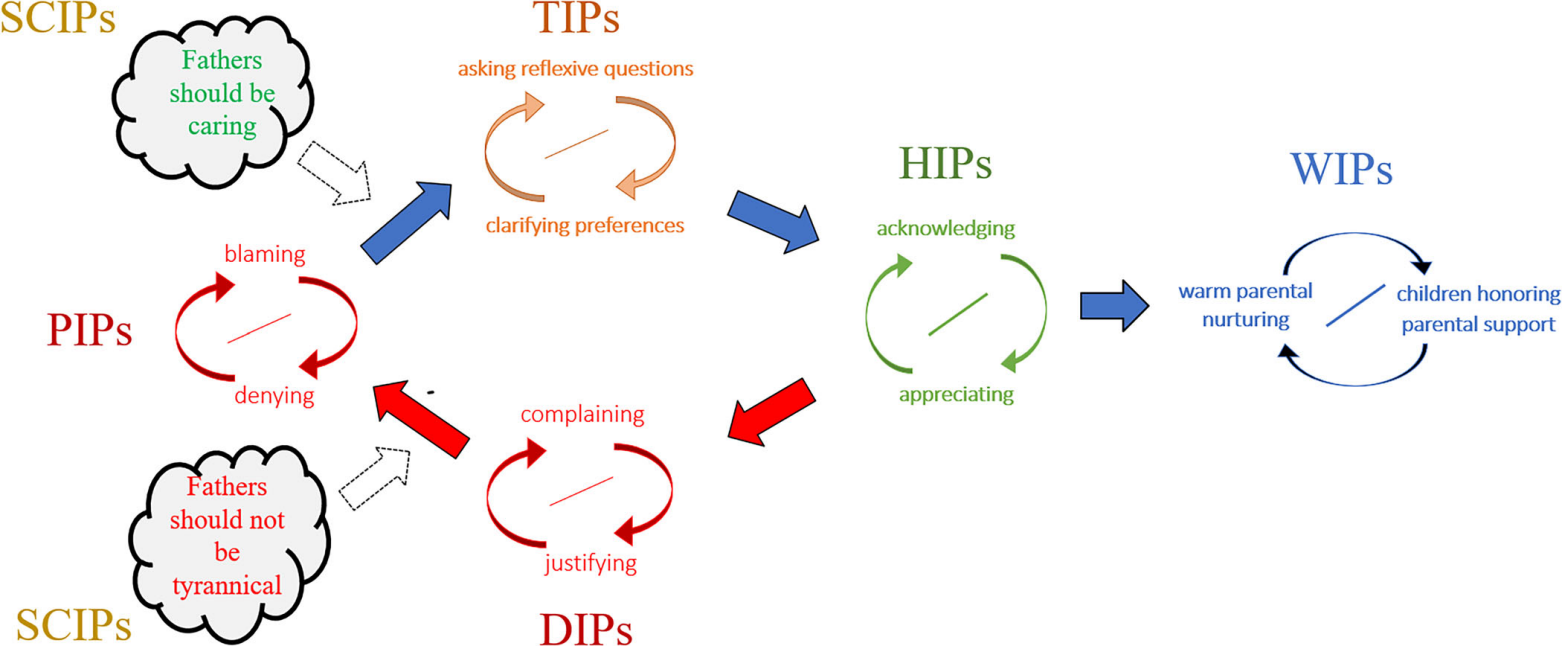
An IPscopic template illustrating movements among different IPs. *Note:* This figure includes six categories of interpersonal patterns: Pathologizing Interpersonal Patterns (PIPs), Deteriorating Interpersonal Patterns (DIPs), Transforming Interpesonal Patterns (TIPs), Healing Interpersonal Patterns (HIPs), Wellness Interpersonal Patterns (WIPs), and Socio‐Cultural Intersonal Patterns (SCIPs). [Color figure can be viewed at wileyonlinelibrary.com]

Figure 1 depicts some possible connections among these different IPs in a generic IPscopic diagram to illustrate dynamic movements within a relationship system. The arching arrows reflect mutual relationship invitations and the central slash indicates their complementarity. Also included in the figure is an example of a possible *Deteriorating Interpersonal Pattern* (DIP) of *complaining coupled with justifying,* which could enable a slip back to the original PIP in this set of examples, as well as possible SCIPs (e.g., normative ideas about parenting) influencing these IPs. We prefer to draw the couplings vertically when a power differential may be operating between the behavioral components in the IPs (e.g. in most PIPs, HIPs, TIPs, and DIPs), and horizontally when the complementarity is smooth and seamless (e.g. in WIPs). SCIPs tend to be in the background and influence IPs with more or less intensity depending on the situation. While every family cycles through all of these different types of IPs at some time in their living together, families prefer (by definition) to spend most of their time in accordance with their own relational hopes (represented with HIPs and WIPs on the right‐hand side of the diagram; a comprehensive description of the IPscope is available in Tomm et al. 2014).

## 4 Seven “M's” of Relational Pain and Seven “R's” for Recovery

We have differentiated seven kinds of relational pain: Misjudging, Misaligning, Misrecognizing, Misappropriating, Mistreating, Mistrusting, and Misgrieving, which we see as sustained by different relational couplings. Although these forms of pain are qualitatively different, they often overlap, sometimes with multiple kinds of pain contributing to a single situation. As such, the prominence of one form of pain can shift into another as the nuances of interaction unfold. At the same time, however, we have the impression that most people experience these forms of relational pain similarly, namely as being trapped in a bind of “I should…but I can't.” This bind is maintained by an ongoing or intermittent PIP in which the interacting participants are immersed. Both parties usually experience significant pain, but the degree of suffering varies according to the situation and the power differential between the participants in the PIP. As a starting point, we usually begin by attending to the more intense pain, which tends to be experienced by the more disadvantaged party.

We use the first‐person singular pronoun (“I”) to highlight the experiential aspect of the relational pain (not to indicate that we see the pain as an individual phenomenon). We acknowledge that pain is typically understood as something that happens “in” individuals. To use a common Wittgenstein formulation, such is the “grammar” of pain (Wittgenstein 1953): something that is “felt” by an “experiencer”—which prototypically corresponds to an individual. While we join and respect those forms of ordinary understanding, we challenge them in therapeutic/inviting ways. As systems‐informed family therapists, we celebrate Minuchin et al. (2007) invitation to go from symptom to system. Starting from the symptom of relational pain, we identify seven systemic descriptions that arise from “I” statements that have the common root of “I should…but I can't because…” These experiential statements correspond to each of these kinds of relational pain:

- I should be able to do more but I can't because I'm a failure.
- I should behave properly but I can't because I don't know how.
- I should accept the other but I can't because it feels wrong.
- I should make my own choices but I can't because I'm being oppressed.
- I should stand up for myself but I can't because I'm powerless.
- I should be able to trust but I can't because I was too deeply hurt.
- I should move forward in my life but I can't because I lost something very dear to me.

From a bioecological standpoint (Bronfenbrenner and Morris 2006), we interpret these “can't because…” statements as expressions of relative disability (Gaete and Gaete 2021). They indicate that individuals come to experience their hopes as hindered by certain relational obstacles. People may not be fully aware of the obstacles nor the corresponding relational hopes they hinder; but they are acutely aware of the pain they feel when something is not right for them. The hoped‐for relationship can be seen as implicit in the type of pain they feel.

It was during a dialogical process among staff therapists and interns at CFTC in Research As Daily Practice meetings (George et al. 2015) that we began identifying different forms of relational pain. We did so by reflecting upon our own disquiet as therapists when addressing various concerns presented by troubled families. An initial focus on common disruptive behaviors in family systems (e.g., aggression) prompted us to formulate the Misaligning pain as “I can't (behave better) because I don't know how to.” This formulation oriented the therapy toward inviting the whole family to learn specific skills that seemed valuable to all family members (e.g., how to manage frustration more effectively). However, when the disruption seemed tied to important identities (e.g., neurodivergent, trans, multicultural), it seemed more coherent and effective to encourage acceptance rather than change. The latter led to the formulation: “I should accept but can't because it feels wrong,” which we labeled as Misrecognizing. We went on to articulate additional types of pain using similar formulations and stopped at seven, mostly because our goal was to outline easily recognizable and common “specimens” of relational pain to guide our practice in working with these families; not to create an exhaustive list of all possible kinds of pain.

Accordingly, we will elucidate seven types of relational pain, outlining for each a prototypical PIP that sustains it, a possible HIP that could displace it, a corresponding relational preference or WIP, and a possible TIP that could help move the interaction from the PIP toward the HIP or WIP. We refer to these examples as prototypical to encourage therapists to customize their relational formulations by attuning carefully to each family's unique situation. We discourage therapists from applying cookbook formulations that might oversimplify the vast variety of family situations that might fit into these categories. In the remainder of this paper, we will describe some common interactional dynamics associated with each kind of relational pain and offer examples of specific reflexive questions within TIPs that might enable recovery toward a family's implicit relational preferences.

## 5 Misjudging and Reappraising

**Figure 2:**
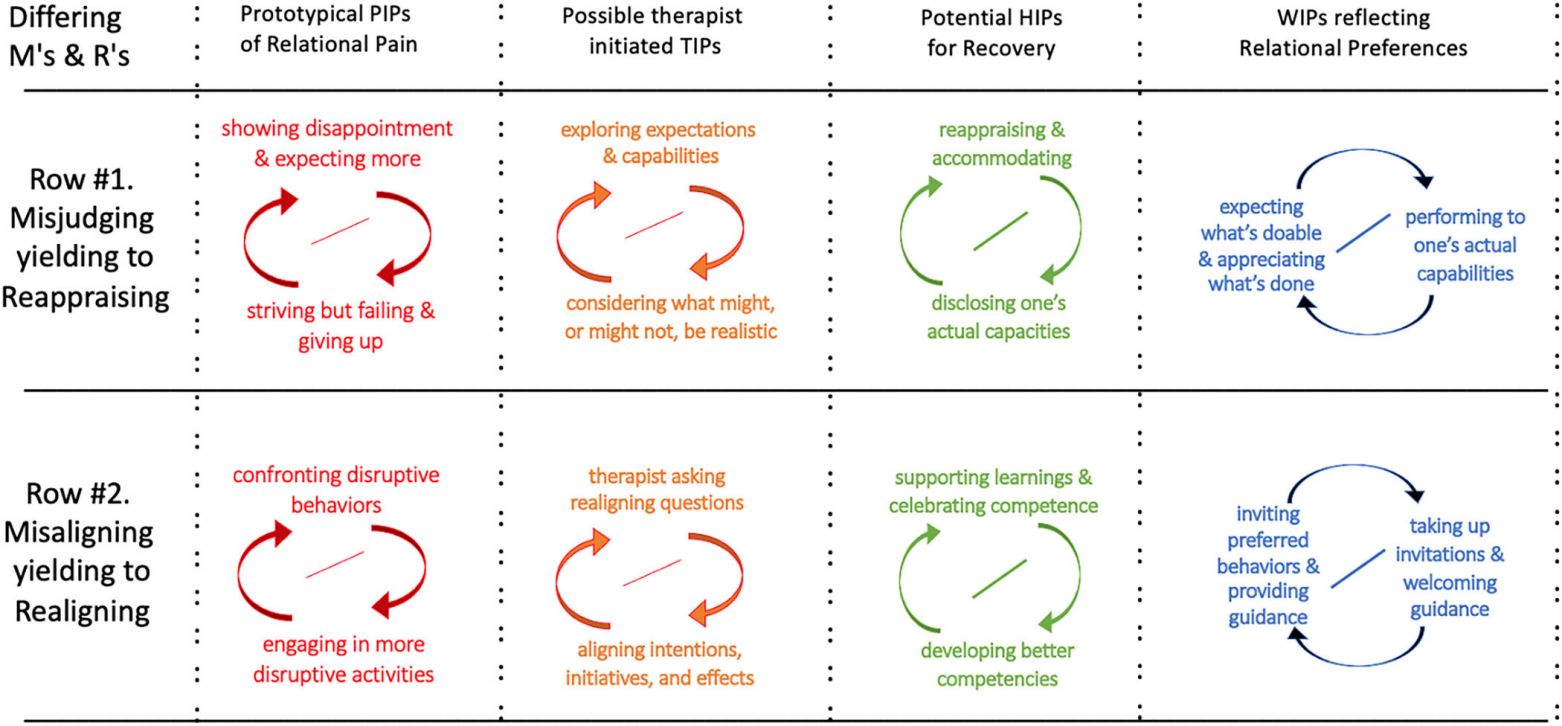
Examples of PIPs, TIPs, HIPs, and WIPs related to Misjudging and Misaligning. *Note:* This figure demonstrates in each column examples of prototypical interpersonal patterns related to Misjudging (Row #1) and Misaligning (Row #2) and their movement toward wellness‐oriented relationships (Reappraising, Row #1, and Realigning, Row #2). [Color figure can be viewed at wileyonlinelibrary.com]

It is quite common for one family member to misjudge the capabilities of another family member and to hold expectations for them that are beyond their ability to fulfill. The failure to meet such unrealistic expectations results in frustration and disappointment for one or both parties, and often leads to relational pain for both. This form of pain can arise regardless of whether the expected capacity is in the physical, intellectual, emotional, volitional, or relational domain. As humans, we implicitly make a multitude of assumptions and assessments of one another in our day‐to‐day interactions, many of which are mistaken and do not match up with what the other is capable of. In other words, we often misjudge other's capacity and when we act according to our misjudgement, relational pain usually emerges. This pain may then become stabilized as an interaction pattern of *showing disappointment and expecting more, coupled with striving but failing and/or giving up,* which recurs again and again as a component of the relationship. This PIP may be seen as part of a series of prototypical IPs related to Misjudging as outlined in Figure 2, Row #1 (and could populate an IPscopic template like that displayed in Figure 1).

Our functioning and capabilities can easily be compromised (Gaete and Gaete 2021) when influenced by substances (e.g., alcohol or drugs), limited by medical disorders (e.g., epilepsy, autism, ADHD, cerebral palsy, traumatic brain injury), or facing major contextual barriers (e.g., institutional, economic, sociopolitical). For instance, people struggling with chemical addiction may find it impossible to stop using, even when they and their loved ones desperately want them to. Similarly, neurodiverse individuals may not respond to social cues in neurotypical ways. And parents require appropriate housing, education, and health systems (beyond just goodwill and parenting skills) to adequately care for their children.

Furthermore, as humans, we tend to internalize other persons' misjudgements of our own capacity (e.g., “I should be able to, but I can't because I'm a failure ‐ there's something wrong with me”). This situation often involves a double form of injustice. First, expecting something of someone who is not capable of delivering (what is expected) is obviously unfair. Second, misjudged persons may not have the means to advocate for themselves and often fall under the influence of an individualizing discourse, resulting in demoralizing self‐blame. The experience of failure may be further intensified as the misjudgment spreads among others upon whom the person depends physically and emotionally (parents, peers, relatives, teachers, health workers, etc.).

Families usually experience enormous relief from the pain of misjudging when they come to recognize that certain expectations are unrealistic, and they find ways to reappraise their expectations. A series of reflexive questions about what might be more realistic could invite family members to reflect in ways that would enable them to engage in such a process of reappraisal: “What is it that you expect from your daughter? …What happens to you when you hear your co‐parent insist on what they think she should do? … Say that was not possible, who in your family would be the first to recognize this? … How could one go about determining what actually might be possible for her? … What are some barriers getting in the way of your daughter achieving…? How much of your expectation depends on your daughter and how much on… (school system, health system…)? (to daughter) What is it like for you when your parents expect you to be able to do this when you can't? … (to all) How can you tell when a reasonable expectation crosses the line to become an unreasonable demand?… How do others (e.g., teachers, elders, neighbors) go about determining who needs to accommodate on what, to maximize her potential?”

Externalizing conversation (White 2007) may also be useful to explore the positive or negative effects of various expectations held by family members: “Where did this idea that she should be able to do this come from? … Did someone tell you this, or is that an assumption that came to you on your own? … In what ways is this expectation helpful or problematic? …When you are under the influence of the idea that she could easily learn how to do it, what are you liable to say or do, that you would not, if you were free of that idea? …What is another idea (about what is more doable) that you could embrace instead?”

Overly ambitious therapists could inadvertently inflict the pain of Misjudging upon their own clients. Family members may experience this pain when their therapists expect them to easily alter their expectations of each other. It may be unrealistic for us as therapists to expect clients to quickly change their unrealistic expectations of others—at least in the short term. We need to remain mindful of our own limitations as well as those of our clients and should be ready to suggest specific referrals to other professionals to conduct client‐centered assessments, to help reappraise their expectations (e.g., a neuro‐psycho‐educational evaluation for a child's learning difficulties) and to seek a consultation or supervision for our work to help reappraise our own expectations of our clients.

## 6 Misaligning and Realigning

Families frequently complain about disruptive behaviors by a certain member of their family. The behavior is not aligned with what is considered acceptable within their local culture. As a result, the person who is disruptive tends to be confronted. A common response to confrontation is more disruption as indicated in the PIP of *confronting coupled with disrupting* (Figure 2, Row #2). The intended effects of a confrontational means to correct disruptions usually do not align with the actual effects, and consequently, significant relational pain emerges.

The disruptive behaviors may include defiance, aggression toward others, self‐harm, impulsivity, inattention, frustration intolerance, “poor” eating habits, and “inappropriate” moods like anxiety and depression. The quotation marks on “poor” and “inappropriate” are intended to highlight that these behaviors are disruptive to someone, whose perception is influenced by some implicit standard of what is appropriate. Parents in pain often account for their child's disruptiveness as the child *not being willing* to behave appropriately. This kind of explanation often activates the criticism or confrontation that begets more disruption. For example, a parent might condemn the child's disruptive behaviors, which may trigger the distraught child to engage in further disruption, which triggers more condemnation, and so forth. An alternative explanation to the “won't behave better” that parents may be open to consider, is that the child “can't behave better” because she has not had sufficient opportunities to learn how to behave properly. The first formulation (“able but not willing”) tends to transform a learning problem into a more complicated moral problem, while the second (“willing but not yet able”) opens space for a joint learning project: the family may be able to re‐align will and skill by creating better conditions for success in learning relevant abilities.

In situations of misalignment family members are often willing to acknowledge that while they may object to the interactive means applied toward behaving appropriately (e.g., parents criticizing, yelling, condemning, etc.), they do not object to the ends or hopes (i.e., everybody in the family agrees that learning to behave respectfully toward one another is both possible and desirable). In other words, there is a discrepancy between the means and the ends. Accordingly, the family members who are most motivated for therapy (often the parents) may accept a description of the pain as someone “can't behave better…because they don't know how.”

A useful focus to address the pain of misalignment is conversations oriented toward *asking realigning questions coupled with aligning intentions, initiatives, and effects* as outlined in Figure 2, Row #2. One place to start could be to co‐construct agreed‐upon goals for therapy by asking questions that help family members coordinate (e.g., name, describe, illustrate) what needs to be learned in order to decrease disruption (e.g., “managing frustration acceptably”); and how such learning could take place (e.g., “exercising patience” and “practicing self‐soothing”). It is assumed that once family members develop the relevant skills (e.g., “building tolerance muscles”), the disruptive behavior generating the misalignment will gradually disappear. Thus, a recovery pathway from misalignment pain could be framed as a collective learning project for realignment; a process in which learners need a learning‐enabling environment (teachers, learning opportunities, supports, etc.).

Reflexive questions separating positive intentions from unintended negative effects are often helpful to enable realignment. Any component of a misalignment PIP (e.g., confronting) may be regarded as an unintended (negative) relational effect that is activated by the complementary component (e.g., reactive disrupting)—not by the positive intentions behind those components. Indeed, the underlying intentions are usually very well‐aligned with people's relational hopes. It is the means that are misaligned with the ends (the intended effects). A series of questions in the TIP could bring forth an awareness of the inconsistency between intended effects and unintended actual effects: “What were you hoping would happen when you yelled at her? (“that she would learn from her mistakes”); When you look back, what actually happened? (“She became more aggressive”); Would you say that was definitely not what you intended? (“Yah*”*) What could you have done differently that might have been more likely to produce the desired effect? (“I don't know”); Who could you ask, that might have some good ideas? (“Maybe I could ask her”); If your daughter understood that your intention was to help her to learn from her mistakes, and not to put her down, how do you think she might respond? (“She'd probably listen more*”*); How do you imagine you might get that message across more successfully?” and so on.

## 7 Misrecognizing and Recognizing

**Figure 3:**
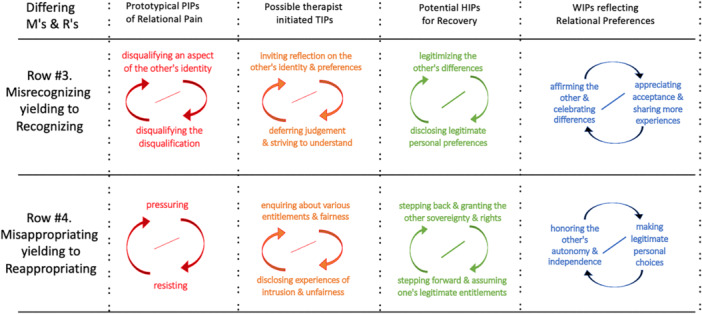
Examples of IPs related to Misrecognizing and Misappropriating. *Note:* This figure demonstrates in each column examples of prototypical interpersonal patterns relating to Misrecognizing (Row #3) and Misappropriation (Row #4) and their movement toward wellness‐oriented relationships (Recognizing, Row #3, Reappropriating, Row #4). [Color figure can be viewed at wileyonlinelibrary.com]

As human beings, our identities are partly shaped by recognition from others (or by its absence) and sometimes by painful misrecognition from others (Taylor 1994). Some families experience themselves not so much as struggling with disruptive behaviors (misalignment), but with *ways of being* that are perceived as problematic. For instance, a teenager identifying as queer can be relationally disruptive to parents who understand gender as binary. In this situation, it is not lack of skill that prompts the pain, but the expectation to live in accordance with others' values which are at odds with one's own values and feels inauthentic. Instead of “I can't … because I don't know how to” the pain here is better formulated as “I can't… because I refuse to accept something I feel is wrong.” It feels wrong for a trans child to comply with their sex assigned at birth, while the parents might see the child as wrong in going against nature and being disrespectful. The parents may reject the child's identity, and the child then rejects the parents' judgments of them. The result is that each party regards what the other party is doing as offensive and inexcusable. Both often find themselves actively disqualifying the other's preferences rather than exploring the background needed for each party to understand and appreciate the legitimacy of the other's values and beliefs. Such disqualifying misrecognition can be experienced as highly threatening, prompting people to overstate their views and resort to extremes to get their “truth” across. Consequently, they may engage in an escalating pattern of mutual disqualification, as depicted in Figure 3, Row #3. Both parties feel that their identity‐making preferences are misrecognized (i.e., dismissed, demeaned, or condemned).

Although misrecognizing situations could be seen as a form of misalignment, we see them as misalignment of a second order, that is, not so much about *perceptions* of preference but about *conceptions* of preference (e.g., clashing identities/worldviews). The disruption is more heavily grounded in the landscape of meaning (misrecognition) than the landscape of action (misalignment). In another example, the members of a couple may have discrepant conceptions about their preferred kind of intimate partnership. For one party, sexual loyalty may be an essential (conceptual) part of the partnership, but only accidental and not essential for the other partner. In a third example, one parent may consider unconditional positive regard as essential to parenting, whereas for the other, this is only a recipe for spoiling a child, and so forth. Misrecognized parties view their pain as stemming, not from any lack of skill (i.e., misalignment), but from fundamental ethical differences (“what you do/want me to do feels wrong to me”).

Therapeutic initiatives here could be focused on helping family members recognize the legitimacy of their respective differences. They are often willing to acknowledge these differences when they feel that what they cherish is not being disqualified and that they are not pressured to value what the other party holds dear. Grounded in a Bringforthist assumption that love involves the full recognition of the other as legitimate in relation to oneself, we often ask family members questions to co‐assess the legitimacy of their identity claims. We see the interruption of mutual disqualification (e.g., judgment, censure, condemnation) as a crucial step toward legitimizing interpersonal differences. In our experience, when people are supported to share their views without immediate critical judgment, they often change the way they express their views—and their views on the other's views.

Deferring immediate judgment opens space for people to share their preferences in a way that is less defensive, less intense, and more truthful for them. This may lead to a prototypical HIP as outlined in Figure 3, Row 3, namely *legitimizing differences coupled with sharing personal preferences*. One way to promote legitimizing differences is through TIPs that separate understanding from agreeing. Therapists may ask questions oriented to acknowledge differences: “What is your perspective on …? …What do you think your partner's perspective on (such‐&‐such) might be? …To what extent could you agree that the views you two have (on this issue) are quite different? …When would it be reasonable to agree to disagree?” This can be followed with attempts to disclose the (implicit) immediate judgment using observer‐perspective questions: “What is your sense of what your partner was thinking when you shared your views?… How did you feel when your partner shared their thoughts about your stance on…?” which invites them into the other's experience (e.g., “Oh, she thinks I'm nuts”) and to the other's experience of one's own experience (e.g., “she feels judged and criticized”). This may be followed by externalizing the judgment as an unintended effect: “Would you say that it was not your intention to disqualify your partner's view? … How would you describe this unintended effect? … Who or what might benefit from this way of seeing things? … How ready are you to explore alternative views that are less likely to come across as disqualifying?” This could be followed by co‐visioning potential benefits that may result from distinguishing understanding from agreeing: “What is the difference between someone agreeing with you as opposed to simply understanding you? … Say it became clear that she did not agree with you but she understood where you are coming from, would you see that as a good thing? … What other good things might come from you feeling less judged and more understood?”

As already noted, we see the deferral of immediate judgment at the core of recognizing TIPs. When the relational pain of misrecognizing seems entrenched and unrelenting, structured exercises to scaffold a clarifying conversation to maximize safety by deliberately delaying interpersonal judgment as in Reflective Listening (Tomm and Acton 2011), or an Internalized Other Interview (Mudry et al. 2015), often help.

## 8 Misappropriating and Reappropriating

While human relationships always entail mutual influences, there are often circumstances where certain influences are experienced as subjugating, restrictive, and/or controlling. Whenever one party persists in exerting such unwelcome influences, they risk misappropriating the autonomy of the other party. Given that most human beings value self‐determination very highly (as showcased in the popular song “I did it my way”), any outside attempt to appropriate one's autonomy may be experienced as a personal threat and result in significant relational pain (“I should be free to have more say about my own life, but I can't”).

A typical response to misappropriation is for the second party to actively resist the unwanted influence. When the former party feels entitled to have that influence (e.g., an adult having authority over a child), and applies more pressure for the latter to comply, this may provoke more resistance, and the relational pain escalates. A common example is a worried parent demanding more obedience from a defiant adolescent: the more the parent insists that the adolescent obey, the more the adolescent resists. Another example (reflecting heterosexism) is an overly entitled male partner insisting on more compliance from his female partner who resists.

The resultant pattern of *pressuring coupled with resisting* is one of the most common PIPs maintaining relational pain among the couples and families that present for therapy (see Figure 3, Row #4). The person who is being pressured usually feels oppressed. To make things worse, over time, this party may begin “caving in” to the struggle, and the PIP may evolve into a more entrenched pathologizing pattern of *dominating coupled with submitting,* facilitated by internalizing the former pattern, now featured as internalized oppression. A wide range of social injustices takes this form of misappropriation and oppression.

Simply giving back what has been unfairly appropriated would obviously be the most appropriate healing initiative in relieving the pain of Misappropriation. However, this is often difficult because the appropriator seldom recognizes the undue entitlement he appears to be exercising over the autonomy of the other party. Therapists could initiate a TIP by opening a conversation to co‐construct greater awareness of how an unreasonable sense of entitlement is being enacted and the ways in which the autonomy of the other is being compromised. Questions to the disadvantaged party about their experience in the interaction may be a useful starting point: “What's it like for you when he …?” This could be followed by interpersonal perception questions to the appropriator to enable greater awareness: “What is your experience of her experience in this situation? … Does she take your words as caring advice, or as you imposing your will upon her? … How much value does she place on making her own decisions?” These kinds of questions may open space for greater accountability and could help deconstruct the *pressuring/resisting* PIP.

Alternatively, Reappropriating TIPs could be more HIP‐focused and be initiated with an embedded suggestion question to the appropriator: “What do you imagine it would be like for her if you expressed your preference for what she does, but then made it clear that it is up to her to decide for herself?” or to the compromised party: “What might he do if you respectfully acknowledged his entitlement to make a request, and yet proactively claimed your entitlement to decide for yourself?” To be consistent, the therapist needs to also respect the autonomy of the family members in responding to his/her questions, lest the therapist inadvertently slip into appropriating the client's freedom to accept or reject the therapist's initiatives to bring forth the Reappropriation of legitimate autonomy and entitlements.

## 9 Mistreating and Revealing

The previous four relational pains may be seen as arising from distortions of underlying good intentions within loving attachments in family systems. Unfortunately, there are times when family members might fall under the influence of less prosocial emotional states and proceed to mistreat other family members. Such states may include fear, anger, rage, resentment, jealousy, disgust, contempt, and so forth. Intense negative emotions or self‐serving attitudes (self‐centeredness, greed, desire for power over others, etc.) can overwhelm family members' inherent tendencies for affiliative behavior and activate impulses to exploit or deliberately hurt others—even using physical aggression, or threats thereof. These negative impulses can be enacted momentarily, recurrently, or in an ongoing manner. In situations where there is a low level of conflict, but one family member holds a resource that another family member covets, there is a risk that the latter might resort to subtle or devious means (e.g., dishonesty, misinformation, or manipulation) to gain access to that resource. Needless to say, abusive behaviors can generate an enormous amount of pain and can leave family members feeling exploited, demeaned, shamed, intimidated, and so forth.

**Figure 4:**
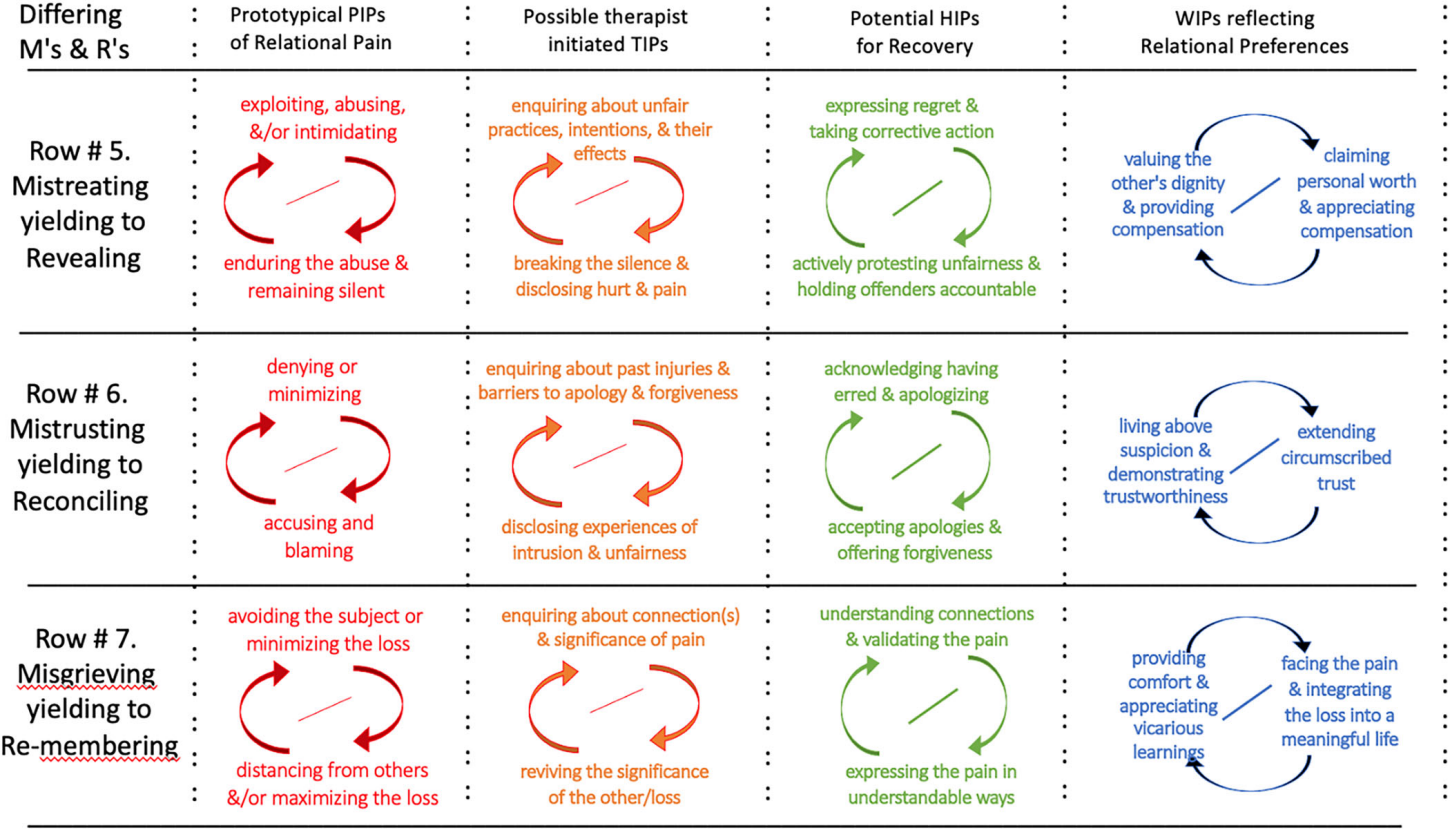
Examples of IPs related to Mistreating, Mistrusting, and Misgrieving. *Note:* This figure demonstrates in each column examples of prototypical interpersonal patterns related to Mistreating (Row #5), Mistrusting (Row #6), and Misgrieving (Row #7), and their movement toward wellness‐oriented relationships (Revealing, Row #5, Reconciling, Row #6, and Re‐membering, Row #7). [Color figure can be viewed at wileyonlinelibrary.com]

It is beyond the scope of this paper to address the myriad forms of mistreatment that can occur in families. What is relevant here is to highlight the relational responses to abusive practices that might inadvertently sustain the mistreatment. Whenever someone is hurt by mistreatment, feelings of resentment and impulses for retaliation tend to arise. If the person who suffered the abuse yields to these reactive impulses and strikes back, escalation can ensue with cycles of emotional or physical violence. Alternatively, the mistreated member may feel too disempowered to fight back. They may seek to escape the painful interaction by disengaging (temporarily or indefinitely). However, when leaving seems impossible or undesirable, the person who is abused may feel trapped and end up submitting to the abuse. One dynamic that contributes to this entrapment is the embarrassment and shame about being mistreated that often orients individuals (and sometimes witnesses) to keep their plight private and secret. Another is the threat of violence if there is any disclosure. This secrecy then contributes to the PIP outlined in Figure 4, Row #5 by maintaining conditions for ongoing mistreatment and minimizing the possibility of others intervening.

A priority in responding to Mistreating is to reveal the injustice and find ways to interrupt the abuse. The unfairness of mistreatment is not always apparent, even to those who are mistreated, especially when they are being deceived and misled by the person engaged in abusive practices. Thus, a series of reflexive questions may be called for to reveal the unfairness and help break the silence. These questions may be addressed to either of the parties involved in the cycle of abuse and to third parties, to co‐construct greater awareness of the injustice. Behavioral effect questions to family members who engage in abuse are especially cogent: “What was the effect on your daughter when you engaged in those (abusive) practices? …What was she thinking? … What was she feeling? … What other consequences resulted for her?”

Family members who engage in abusive behavior may be unaware of the extent of the painful effects and harm they are inflicting. Creating conditions of safety for those who are being abused to make impact statements (if they wish to do so), potentially provides important feedback for others who engage in abusive practices. Interpersonal perception questions can also help disclose underlying intentions: “What do you imagine she thought you had in mind when you …? …What were your intentions at the time?” These could be followed with experience of experience questions which could invite empathy from the person displaying abusive behaviors: “What was your experience of her experience when this was happening? … What is your experience of her experience as she is sharing this right now?” Further reflexive questions could gradually invite feelings of regret, which could eventually open space for a possible apology and some restorative action.

Interrupting ongoing abuse can, at times, be extremely difficult. On occasion, it may require going beyond therapeutic means, as when therapists take unilateral action by calling local authorities. Deconstructing mistreatment therapeutically usually requires heavy doses of accountability. Unfortunately, the process of holding those who engage in abuse accountable can itself be experienced by them as mistreatment, especially when there is little awareness of the harm they have perpetrated. In addition to the questions that can facilitate awareness, externalizing questions can go some distance in opening space for moving toward more fairness and restorative justice. If family members can see themselves as separate from the abusive practices they have engaged in, they can more readily embrace intentions that are grounded in compassion rather than resentment.

## 10 Mistrusting and Reconciling

Family members often understand their pain as the aftermath of past mistreatment. The behavioral disruptions may no longer be taking place, but the residual effects keep rippling on. Perhaps a partner broke a marital vow in having an affair, or a guardian who ought to have been protective was abusive. In these situations, family members may feel that the other is no longer to be trusted, and they continue to be wary because of their woundedness. A formulation of “I can't trust because I have been damaged by someone” usually fits. This mistrusting form of pain may be intensified by the absence of any acknowledgment of the injury (an additional wound of silence). Any dismissal (especially from those perceived as being abusive) brings forth further resentment and mistrust for those who see themselves as injured, often perpetuated by a pattern of *denying or minimizing coupled with accusing and blaming* (Figure 4, Row #6). In such situations, family members are usually willing to acknowledge that their pain revolves around the issue of trusting. The other family member may also feel hurt in not being trusted after they have reformed their ways. But when they insist that they ought to be trusted again, the mistrust tends to deepen.

A pathway toward rebuilding trust could be initiated by inviting a series of acknowledgments. Therapists themselves may start by simply acknowledging and normalizing the difficulty in trusting, as voiced by a family member who, for instance, feels betrayed. Then, a series of reflexive questions could facilitate acknowledging a connection between perceived trustworthiness and trusting: “How do you see trust related to trustworthiness?… Is it possible to trust a person who is not perceived as trustworthy?” Acknowledging the importance of privileging perception over objective statements often helps people to validate the experience of the other—their wounds, their pains—rather than an assumed objective truth. A suitable next step in the conversation could be to ask questions that may bring forth the importance of demonstrating trustworthiness before expecting trust to recover. This may reflexively motivate a prior offender to demonstrate that they could be trusted again: “If you had opportunities to demonstrate your trustworthiness, would you take advantage of them? … If you wanted to show more trustworthiness, how would you go about doing so?” Introducing a distinction to separate *doers* from *doings* is often helpful here, making it easier for those who have engaged in mistreatment to acknowledge their wrongdoings. Such acknowledgments are usually very meaningful to hear for those who have been offended.

Encouraging family members to go further and extend genuine apologies is a way for them to implicitly acknowledge that the person at the receiving end of the abuse deserved better. If the person engaged in abusive behavior can go beyond the apology by taking corrective and restorative action, it becomes easier for the person suffering the abuse to recognize the emergent trustworthiness and begin moving into the reconciling interaction patterns in the HIPs and WIPs in Figure 4, Row #6. When there is significant discrepancy in the perception of injuries, it is often useful to ask questions to enable enhanced interpersonal perception e.g., asking: “Say your partner thought that you felt what happened to her was not that offensive, or had not really been very hurtful, how do you imagine this may affect her ability to trust you?”.

Parallel work with family members who have been abused may also be warranted. While the mistrust may initially be helpful in supporting self‐protectiveness, the associated resentment in having been hurt could extract considerable cost to a person's wellbeing. Bifurcation questions to invite reflection upon when the mistrust might still serve a purpose and be useful, as opposed to when it might be more problematic than helpful, may be a good place to start. If the person who suffered abuse expresses hopes to regain trust and be freed from resentment, a conversation about forgiveness could be introduced. Once again, separating doers from doings can be helpful here: it is the person (who committed the offense) that is forgiven, not the behavior of the offense. The interpersonal pattern of *apologizing coupled with forgiving* is one of the most powerful healing dynamics to enable reconciliation.

## 11 Misgrieving and Re‐Membering

Not always, but typically, the pain of misgrieving results from traumatic events in which nobody can be blamed. There is a wound, it is incredibly painful, but there is no perpetrator. The pain may be the result of an unfortunate accident rather than an unjust event. The death of a loved one is a classic example. When a family or a family member is grieving, they may come to view their pain as “I can't (live in the way I'm used to, or prefer to), because I have lost someone essential for doing that.” This form of pain may be aggravated when it triggers discomfort in significant others and activates minimizing responses from them: “Don't you think it's time to let it go and get on with your life?” Often, these others do not have sufficient understanding to appreciate the significance of the loss, or do not know how to soothe the hurt. Or perhaps they are influenced by the stages of a grief model with the goal of accepting the loss. Whatever the reason, this minimizing may be experienced as dismissive by the grieving person which often brings forth a complementary process of maximizing the loss instead, which only increases the minimizing (now perhaps adding distancing, with further loss of connection). We illustrate this complication in the PIP in Figure 4, Row #7.

Families with members dealing with the pain of misgrieving are usually willing to acknowledge that the original pain was legitimate and valid, and deserved proper expression and consideration. The person may have been granted time and space to share how they experienced their loss. However, it is often later when they are expected to accept the loss and “let go” that the pain gets worse. Realizing that memories of the lost relationship never need to be abandoned, and could be actively nurtured instead, usually comes as a huge relief. For example, the person who is no longer breathing can remain a full‐fledged member of the person's family or social network and can be drawn upon anytime in one's imagination. Reflexive questions may facilitate such a shift: (to the minimizer) “What is your understanding about why this connection is so significant for … (the bereaved person)? … What might the person who is no longer breathing say about the importance of the relationship?” And to the bereaved person: “Tell me more about her (the deceased person) and your relationship with her? …In what way is your pain a testimony of the strength of your relationship with her? …What would she say about this situation?”, leading to a better understanding of how the pain speaks to what people hold dear in their hearts and should not be lost.

By calling this process re‐membering we join the many cultures that have considered the heart as the center of memory. Indeed, the ancient Latin word “recordari” meaning “to remember” literally denotes passing it again (re‐) through the heart (cordis). Drawing upon White's (1988) article *Saying Hullo again*, therapists may invite family members to describe themselves through the internalized eyes of the deceased person: “If you were seeing yourself through the eyes of your loved one, what would they be noticing about you and your pain that they would appreciate?… What difference would it make to your pain if you were accepting of this in yourself right now?… Why would you say it is important for you that their knowledge about you does not get lost?” The voice of the lost member may in this way continue to console the bereaved person and their relationships: “Would you like to introduce others to this way of understanding yourself and your relationship with the lost one? … How would you go about doing so? … If others came to see you/this issue more in this preferred way, what might be different for you/your world?”

These conversations can facilitate people re‐connecting with both actual others and internalized others, supporting an integration of loss and pain in ways that enrich, rather than impoverish their lives. Outsiders may feel inspired and invited to support this process of re‐membering and reconnecting, and may learn something themselves about how to integrate suffering to enrich a person's life (see Figure 4, Row #7).

## 12 Shifting From One Pain to Another

We have described seven kinds of relational pain which, as different as they may be, are at the same time intricately interconnected and easily shift to flow from one to another. Take, for example, a situation where a teenaged son is struggling with online schooling during a pandemic, having difficulty understanding some maths concepts. The marks on his report card drop and his parents start asking questions about his study habits, which he is unable to answer and leave him feeling like a failure. As a result, the pain of Misjudgment arises in the parent‐child relationship.

The father decides to take control of the situation by arranging a series of appointments with a math tutor; the son now feels not only like a failure but humiliated and that his autonomy has been compromised. He protests and rebels against the arrangement. Consequently, the pain of Misappropriation enters the scene. The mother tries to help her son see the value of the tutor but ends up criticizing the son for not accepting the father's plan. The son starts shifting from outer protest to inner protest to resist his parents' efforts to impose their outer controls: he stops doing his homework. The mother's good intentions of helping the son to cooperate are not aligned with the effects on him, and the pain of Misalignment arises.

To escape the increasing parental pressure, the son disregards schoolwork altogether and becomes focused on hanging out with his friends instead. This leads his parents to engage in Misrecognizing what is important to their son by insulting his friends (“those lazy youtubers you hang out with”) and the son disqualifies the parents as uncaring. As the situation deteriorates, father and son begin calling each other names, which raises the pain of Mistreating. Father and son escalate into some physical altercation, to the point that the son shows wariness of his father and avoids all contact with him. The pain of Mistrust has entered to further complicate the situation. In the days and weeks that follow, both the father and son experience the pain of Misgrieving a loss of the closeness and connectedness they once had.

This family could present for therapy anywhere along this progression. We submit that some familiarity with these diverse kinds of relational pain and their relevant IPs could help a therapist attune and respond more precisely to the pain and more likely bring forth relevant therapeutic possibilities. Much depends on how the members of the therapy system (therapist plus family) make sense of the most significant relational pain family members are experiencing and struggling with in the moment. Nonetheless, we offer these seven differing TIPs as alternative pathways to help families step out of specific PIPs and into preferred HIPs and WIPs.

## 13 Concluding Comments

In the renowned depiction of Purgatory in The Divine Comedy by Dante, love is seen as the central driving force behind human behavior. When properly directed, love results in virtue (“God”), but if misdirected, love leads to sin. In the first part of his epic narrative poem, Inferno, Dante described the seven deadly sins as seven forms of misguided love, and prescribes distinct penances aimed at transforming sinful love into virtuous love.

Similarly, we consider the seven “M's” of relational pain to be seven examples of misguided loving in family relationships. Our job as therapists is to bring forth ways that family members might find their way back to more wholesome loving. We draw upon Maturana and Verden‐Zoller (2009) definition of love as the domain of interactions in which the other arises as legitimate in relation to oneself. Acknowledging the legitimacy of the other as different from oneself is arguably one of the most loving initiatives a person can take in relation to another. As therapists informed by Bringforthist Therapy (Tomm et al. 2025), we strive to implement this perspective in our practice by consistently treating our clients with respect and encouraging the members of the families we work with to become more affirming and acknowledging of one another.

Just as Dante went on to identify other sins, we expect other kinds of relational pain to be identified as systemic therapists become more skilled in differentiating additional relational patterns. All seven “R” TIPs described in this paper involve acknowledging and accepting otherness by conversationally bringing forth family members' relational hopes. Through these TIPs, then, relational pains (i.e., PIPs) can be transformed into *relational preferences* (i.e., HIPs and WIPs). Put differently, engaging in “R” TIPs helps create momentum by prompting collective acknowledgment of relational pains, which in turn promotes acknowledging the relational preferences that implicitly and inevitably exist behind them; for there would be no pain if we did not have hopes and aspirations. When we focus on the particulars of offering “R” TIPs, we find ourselves, again and again, asking reflexive questions as a way to scaffold micro‐acknowledgments, which create conditions for small steps toward transforming pain into preference, effectively traversing new terrain. And this is probably how words in therapy have power: they pave our way back to virtuous loving.
